# Case Report: Midgut malrotation presenting as left-sided appendicitis with secondary omental torsion: a diagnostic challenge in a child

**DOI:** 10.3389/fped.2025.1728905

**Published:** 2026-01-12

**Authors:** Han Yao, Puneng Ma, Ruiqi Gao, Chenghao Zhanghuang, Xin Yi

**Affiliations:** 1 Department of Ultrasound, Kunming Children’s Hospital, Kunming, China; 2 Department of General Surgery, Kunming Children’s Hospital, Kunming, China; 3 Department of Urology, Kunming Children’s Hospital, Kunming, China

**Keywords:** left-sided appendicitis, midgut malrotation, omental torsion, pediatrics, ultrasonography

## Abstract

**Background:**

Omental torsion is an uncommon cause of acute abdomen in children and is usually secondary to other intra-abdominal pathology. Congenital midgut malrotation can alter anatomic relationships and lead to atypical presentations. This case report aims to highlight that congenital malrotation should be considered when left-sided appendicitis is suspected, especially when omental abnormalities are present.

**Case presentation:**

We report a 12-year-old child who presented to a tertiary children's hospital in Southwest China with more than 48 h of abdominal pain and 24 h of fever after heavy intake of indigestible meat-based snacks. Initial ultrasound showed diffuse omental thickening in the left abdomen. Repeat ultrasound revealed a hypoechoic avascular area in the perisplanic omentum, raising suspicion of omental torsion. Preoperative computed tomography demonstrated left-sided ileocecal localization consistent with midgut malrotation and features of appendicitis with localized peritonitis. Laparoscopy confirmed purulent peritonitis, a left-sided perforated appendix with abscess, and torsion of the greater omentum near the splenic pole. Appendectomy with peritoneal lavage and drainage was performed, while the omentum was managed conservatively because of gross contamination and the absence of overt necrosis. Postoperative ultrasound showed preserved omental perfusion and confirmed malrotation without volvulus. The patient recovered uneventfully and remained symptom-free during outpatient follow-up.

**Conclusions:**

When children present with left-sided appendicitis and omental abnormalities, congenital midgut malrotation should be considered early in the diagnostic process to optimize preoperative planning. Ultrasound is invaluable for detecting omental changes, whereas an upper gastrointestinal contrast study remains the reference test for confirming malrotation in stable patients. This case underscores the importance of close collaboration between pediatric radiologists and surgeons in managing atypical acute abdomen.

## 1 Background

Omental torsion leading to omental infarction is a relatively rare condition, categorized into primary and secondary types, with the secondary form being more prevalent (1). Secondary omental torsion typically arises from underlying abdominal pathologies, such as appendicitis or other inflammatory processes, and is often identifiable (2). The greater omentum, a large apron-like structure composed of fatty and areolar tissue, is prone to torsion due to its mobility and variable attachments (3). This condition predominantly occurs on the right side due to the anatomical length of the right omentum.

The clinical presentation of omental torsion can be deceptive, often mimicking other acute abdominal conditions like appendicitis, leading to misdiagnosis (4). Pain is initially diffuse due to the stimulation of omental autonomic nerves and becomes localized as ischemia progresses and necrosis sets in. Ultrasound imaging is a valuable tool for detecting omental abnormalities, including omental thickening, increased echogenicity, and the characteristic “whirlpool sign.” However, differentiating omental torsion from other conditions can be challenging, especially when coexisting pathologies are present (5).

Congenital midgut malrotation, a developmental anomaly of intestinal rotation and fixation, can profoundly alter the anatomical relationships within the abdomen. These changes may predispose to atypical localization of the appendix and to unusual patterns of omental involvement. This case highlights the diagnostic complexity of secondary omental torsion in a pediatric patient with midgut malrotation and underscores the importance of a high index of suspicion and comprehensive imaging for accurate diagnosis and management in a tertiary pediatric center.

## 2 Case report

A 12-year-old child presented to a tertiary pediatric referral center in Southwest China in May 2024 with more than 48 h of abdominal pain and 24 h of fever. Symptoms began after heavy intake of indigestible meat-based snacks. The pain was constant and non-radiating, without a clear relationship to movement or meals. The child had reduced appetite, malaise, and loose stools but no vomiting and maintained normal urine output. Intermittent fever peaked at 38.7°C and recurred despite antipyretics administered at a local facility.

On admission, the working diagnosis was acute abdominal pain with suspected peritonitis, and laboratory tests and abdominal ultrasound were arranged.

### 2.1 Physical examination

The child was febrile (38.3°C) with tachycardia (108 beats/min) and a respiratory rate of 21 breaths/min. The general condition was stable. Abdominal examination revealed guarding and generalized tenderness, most prominent in the left lower quadrant with rebound tenderness. Bowel sounds were present.

### 2.2 Initial diagnostic investigations

**Figure 1:**
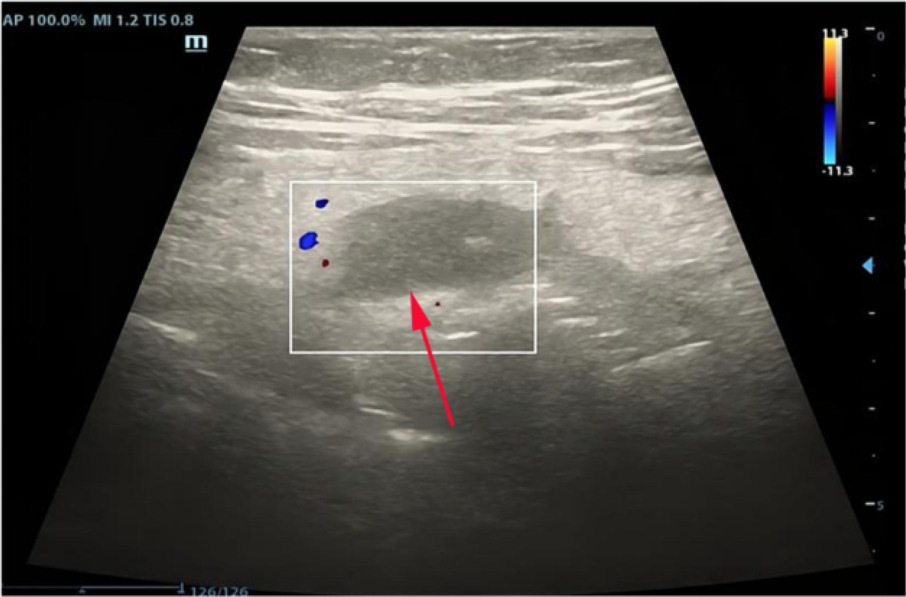
Initial abdominal ultrasound in the left lower quadrant. The greater omentum appears thickened with an internal hypoechoic avascular area (arrow), raising early suspicion of omental ischemia in an atypical left-sided acute abdomen.

Baseline abdominal ultrasound demonstrated marked thickening of the mesentery in the left lower quadrant and lower abdomen. The appendix was not clearly visualized or confidently identified as abnormal (Figure 1).

### 2.3 Follow-up ultrasound findings

Because of persistent pain and fever, repeat color Doppler ultrasound was performed the next day. It showed diffuse mesenteric and omental thickening predominantly on the left side, and a hypoechoic avascular area within the perisplenic omentum, strongly suggestive of omental ischemia and torsion.

**Figure 2:**
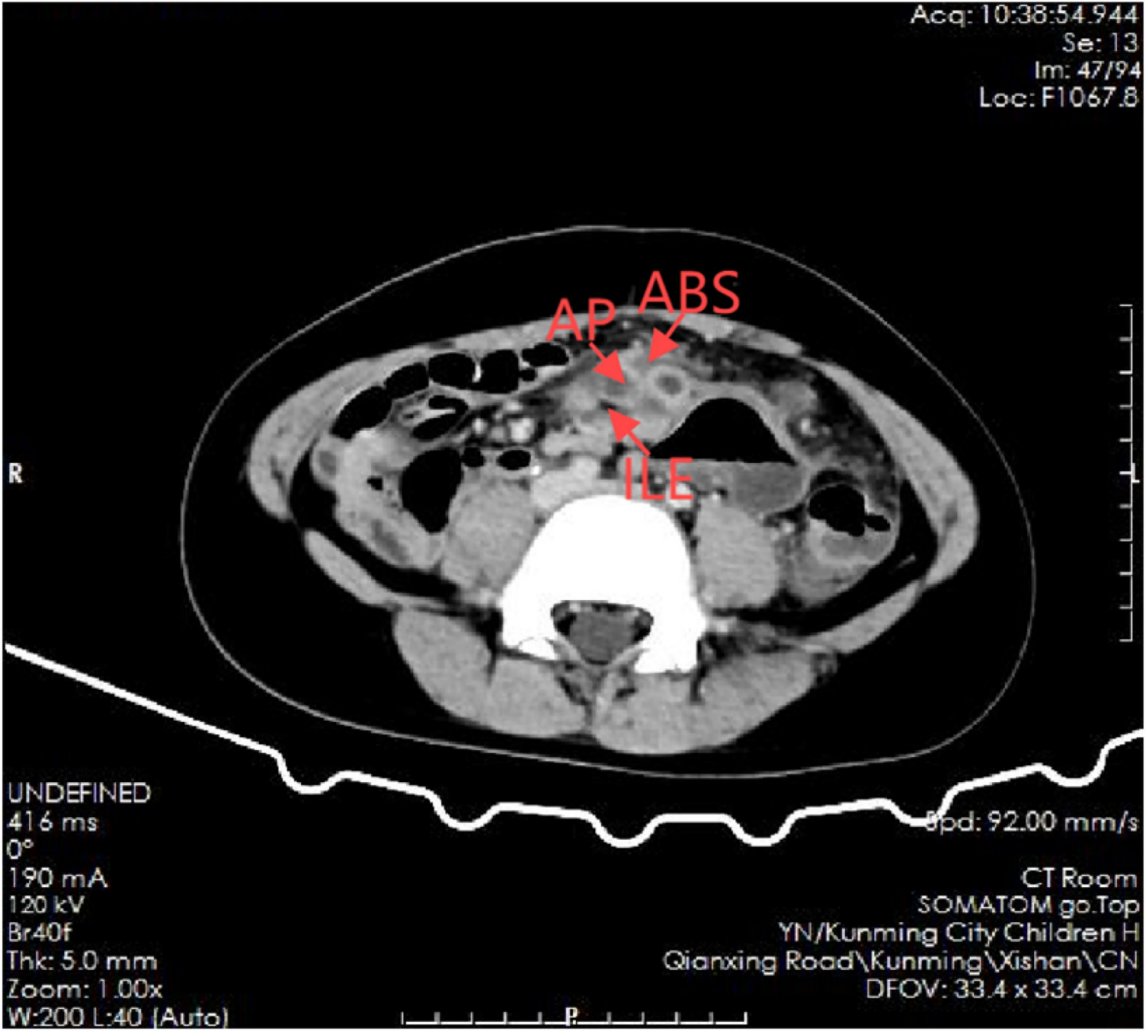
Contrast-enhanced CT showing a left-sided ileocecal region (arrow) and inflammatory changes consistent with left-sided appendicitis. AP, appendix; ABS, abscess; ILE, ileocecal region.

### 2.4 Preoperative CT findings

Preoperative contrast-enhanced CT demonstrated abnormal left-sided positioning of the ileocecal region, consistent with midgut malrotation, without evidence of midgut volvulus. Mild left-sided peritoneal thickening and a small volume of ascites were present. The appendix was identified in the left mid-abdomen with surrounding inflammatory changes, suggestive of appendicitis with localized peritonitis (Figure 2).

### 2.5 Intraoperative findings

**Figure 3:**
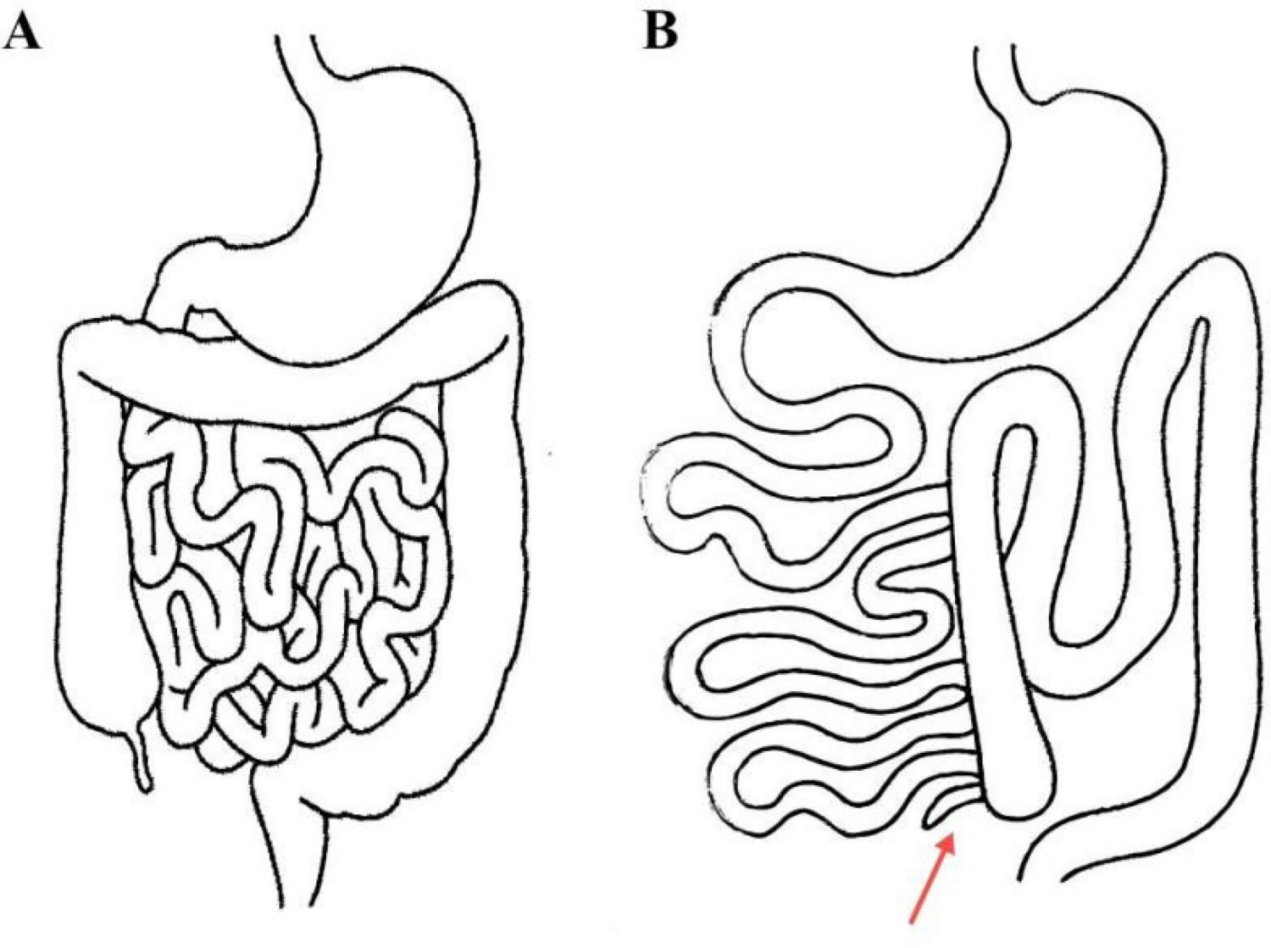
Schematic anatomy. **(A)** Normal intestinal rotation with the ileocecal region in the right lower quadrant and freely mobile greater omentum. **(B)** Midgut malrotation in the present case showing a left-sided ileocecal region and appendix (red arrow) with adjacent greater omentum in the left abdomen.

Laparoscopy revealed purulent ascites. The greater omentum had migrated into the left lower abdomen and partially enveloped the bowel. Omental torsion was identified near the lower pole of the spleen. A left lower quadrant abscess contained a perforated, inflamed appendix. After confirmation, appendectomy with peritoneal lavage and drainage was performed. The ileocecal region was located in the left lower abdomen and was densely adherent to the sigmoid colon, omentum, and abdominal wall. Given the severe contamination and the lack of obvious omental necrosis, no additional procedure for the omentum was undertaken at the index operation. A schematic illustration of the intraoperative anatomic configuration is provided in Figure 3.

### 2.6 Postoperative diagnosis

The final diagnosis was congenital midgut malrotation with a left-sided appendix, complicated by perforated appendicitis, purulent peritonitis, and secondary omental torsion, without midgut volvulus.

### 2.7 Postoperative course

The child received intravenous ceftriaxone and metronidazole for 7 days. Abdominal pain and fever resolved, inflammatory markers normalized, and bowel function recovered. The patient was discharged on postoperative day 10 in good condition.

**Figure 4:**
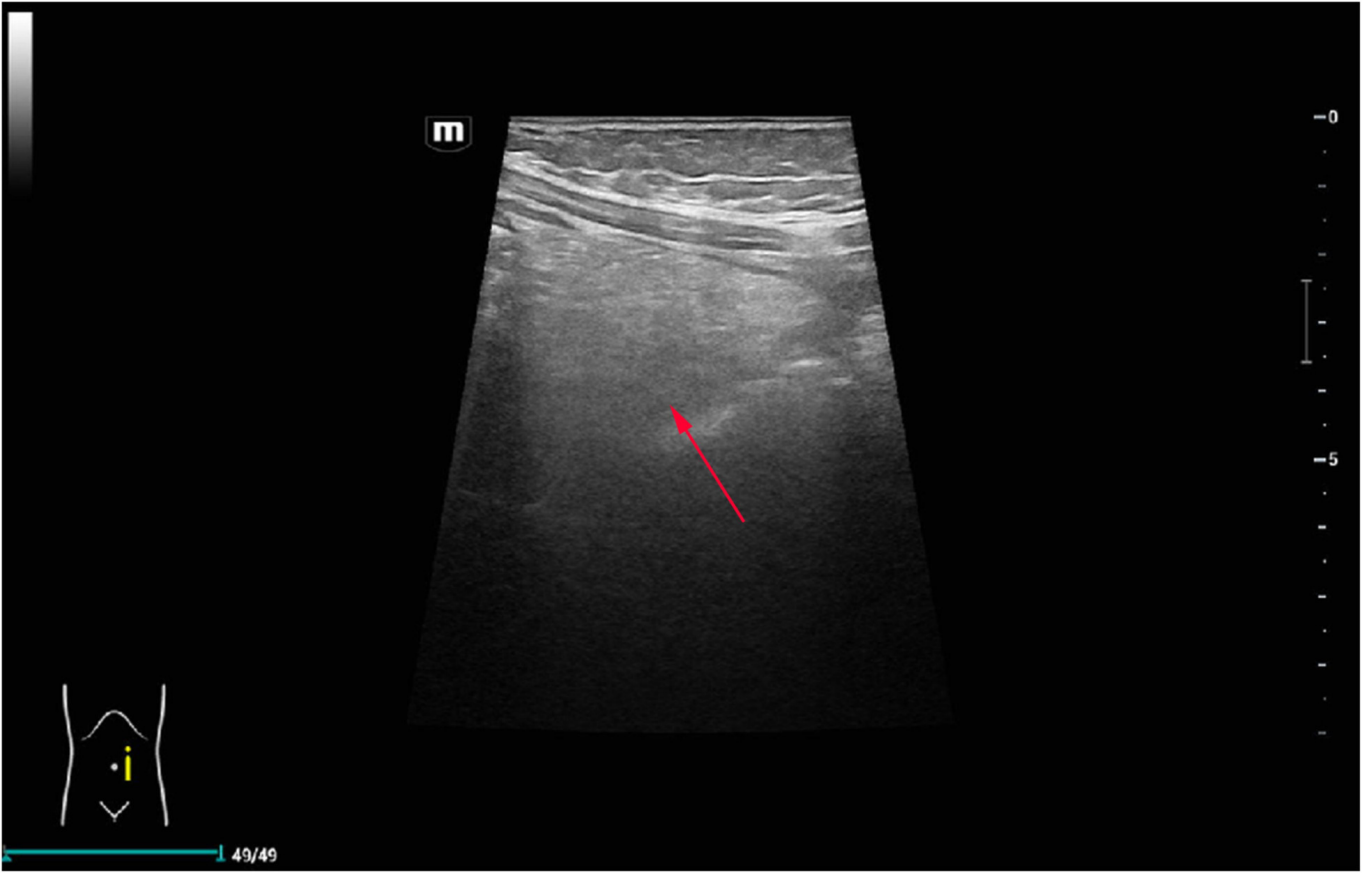
Postoperative follow-up Doppler ultrasound. The previously thickened left-sided omentum demonstrates restored internal blood flow (arrow) and more homogeneous echotexture, indicating resolution of the secondary torsion without infarction; persistent malrotation is noted without volvulus.

Follow-up Doppler ultrasound one week after surgery confirmed persistent malrotation without volvulus and showed preserved perfusion of the previously thickened omentum, indicating spontaneous resolution of the torsion without infarction (Figure 4). The patient remained asymptomatic at 6-month follow-up.

### 2.8 Diagnostic reasoning and missed preoperative diagnosis

Although preoperative CT suggested left-sided ileocecal localization compatible with congenital midgut malrotation, the working diagnosis focused on diffuse peritonitis and suspected omental pathology owing to the prominent inflammatory changes on ultrasound and the child's rapid clinical deterioration. In retrospect, malrotation should be framed as the primary anatomic substrate, with left-sided appendicitis and secondary omental torsion as downstream consequences.

In emergency settings, ultrasound and CT are typically obtained first. When the patient is hemodynamically stable, an upper gastrointestinal (UGI) contrast study remains the reference test for diagnosing malrotation with or without volvulus. In this emergency scenario, a preoperative UGI series was not performed, which limited the level of anatomic certainty before surgery. Table 1 summarizes the sequential diagnostic reasoning process from initial clinical suspicion through imaging, operative findings, and final diagnosis.

**Table 1:** Chronological diagnostic reasoning and management pathway in a child with midgut malrotation, left-sided perforated appendicitis, and secondary omental torsion.

Step	Time point/stage	Key clinical & imaging findings	Diagnostic reasoning (What we thought)	Management decision (What we did)
1	Initial presentation (ED admission)	12-year-old child with >48 h abdominal pain and 24 h fever; pain started after heavy intake of indigestible meat-based snacks; maximal tenderness and rebound in the left lower quadrant; bowel sounds present	Suspected acute peritonitis with a left-sided inflammatory focus; typical right-sided appendicitis pattern not present	Admitted with a working diagnosis of “abdominal pain; acute peritonitis?” and ordered laboratory tests and abdominal ultrasound
2	First ultrasound	Marked thickening of the mesentery and greater omentum in the left lower quadrant and lower abdomen; no confidently thickened appendix identified	Favored “left-sided omental/mesenteric inflammatory changes,” but appendicitis not confirmed; atypical acute abdomen	Continued close clinical observation and planned repeat imaging due to persistent symptoms
3	Follow-up ultrasound (after 1 day of persistent symptoms)	Repeat color Doppler US: diffuse mesenteric and omental thickening, predominantly on the left; thickened perisplenic omentum with a hypoechoic, avascular patch	New key sign of suspected omental torsion → high suspicion of secondary omental torsion	Listed “omental torsion + acute peritonitis” as major diagnostic consideration; decided to perform CT to evaluate bowel position and related pathology
4	Preoperative CT	CT confirmed the ileocecal region in the left lower abdomen; no midgut volvulus; localized inflammatory changes and small-volume ascites	Established: (1) left-sided ileocecal region → congenital midgut malrotation; (2) left-sided appendicitis with localized peritonitis; (3) omental involvement explaining US findings as secondary omental torsion	Reached a high preoperative suspicion of “left-sided appendicitis with secondary omental torsion on the background of malrotation”; planned emergency diagnostic laparoscopy.
5	Laparoscopic exploration	Purulent ascites; greater omentum migrated into the left lower abdomen, enveloping bowel; torsion of the omentum around the lower pole of the spleen; perforated appendix within a left lower quadrant abscess	Confirmed: congenital malrotation → left-sided cecum and appendix → perforated left-sided appendicitis + abscess → contiguous greater omentum with secondary torsion	Prioritized sepsis control: performed appendectomy with lavage and drainage; due to gross contamination, dense adhesions, and absence of frankly necrotic omentum, no formal omental resection or detorsion was attempted
6	Early postoperative course	Intravenous ceftriaxone + metronidazole for 1 week, plus ulinastatin; abdominal pain and fever resolved, inflammatory markers normalized, bowel function returned; discharged on postoperative day 10	Clinical response confirmed that the initial operative strategy was effective and infection was controlled	No immediate Ladd procedure; planned clinical and imaging follow-up instead
7	Early postoperative ultrasound follow-up (∼1 week)	Doppler US: ileocecal region still located in the left abdomen (persistent malrotation) without volvulus; left abdominal greater omentum less thickened with homogeneous echotexture and preserved internal blood flow	Indicates that the omental torsion had resolved without infarction; malrotation persists as a stable anatomic substrate rather than an acute event	Continued conservative management with documentation of malrotation and explanation to the family about future risk and the need for vigilance
8	Mid-term outpatient follow-up (∼6 months)	Patient asymptomatic with no recurrent abdominal pain or obstructive symptoms	Supports that left-sided appendicitis and secondary omental torsion were successfully treated in a single operation and that malrotation is currently silent	Recommended long-term follow-up and advised that any new abdominal pain should prompt reassessment and consideration of elective Ladd procedure

## 3 Discussion

Omental torsion is rare in children and most often occurs as a downstream event secondary to other intra-abdominal pathology. In our patient, congenital midgut malrotation—without volvulus—established an atypical left-sided ileocecal position that predisposed to left-sided appendicitis and localized peritonitis; the resulting inflammation likely triggered secondary torsion of the adjacent greater omentum. This causal hierarchy reconciles the imaging and operative findings and also explains why the initial ultrasound, although sensitive to omental abnormalities, did not immediately reveal the underlying anatomic variant. The comparison summary table of primary and secondary greater omentum torsion is shown in Table 2.

**Table 2:** Clinical and imaging distinctions between primary and secondary greater omental torsion.

Feature	Primary omental torsion	Secondary omental torsion
Underlying cause	Idiopathic; anatomical variants (redundant omentum, focal fat accumulation)	Occurs secondary to another intra-abdominal pathology (e.g., appendicitis, hernia, tumors, adhesions)
Typical age/population	More common in adults; rare in children	Relatively more frequent in children when torsion is triggered by focal inflammation
Usual location	Predominantly right-sided greater omentum	Corresponds to site of underlying pathology; may be left-sided or atypical
Imaging findings (US/CT)	Focal, right-sided, heterogeneous fatty mass with concentric or whirl-like pattern; no clear adjacent inflammatory source	Omental thickening or “whirl” adjacent to an identifiable inflammatory focus (e.g., inflamed appendix or abscess)
Management	Often surgical resection; laparoscopic omentectomy increasingly used	Management directed at primary pathology; omental resection or detorsion considered if necrotic, otherwise conservative management may be feasible
Prognosis	Good with timely diagnosis and treatment	Depends on control of underlying disease and extent of omental involvement

To our knowledge, this combination of congenital midgut malrotation, left-sided perforated appendicitis, and secondary omental torsion has rarely been reported in children.

Mechanistically, malrotation alters cecal and small-bowel fixation, narrows the mesenteric base, and reconfigures the spatial relationship between bowel and omentum. These changes can facilitate tethering or torque on the omentum in the presence of adjacent inflammation (e.g., appendicitis), predisposing to secondary torsion. A closely analogous presentation has been described by Emanuwa et al., where a left-sided cecum was found tethered by torted omentum at laparotomy, supporting the same causal sequence proposed here (6).

From an imaging perspective, both ultrasound and computed tomography (CT) play essential roles in the diagnosis and evaluation of omental lesions. Ultrasound can delineate the characteristic echogenic thickening of the greater omentum and is particularly advantageous in identifying focal hypoechoic regions with absent internal Doppler signals. These features are critical for distinguishing the nature of omental pathology. For instance, in cases of inflammatory myofibroblastic tumor (IMT), ultrasound may reveal a hypoechoic mass, consistent with the typical sonographic appearance of omental lesions (7). However, when the child is hemodynamically stable, an upper gastrointestinal (UGI) contrast study remains the reference investigation for establishing the diagnosis of malrotation (with or without volvulus) (8). In our emergency setting, a preoperative UGI was not obtained, which is a recognized limitation that can reduce anatomic certainty before surgery.

The Ladd procedure is widely regarded as the standard treatment for intestinal malrotation. It entails division of the Ladd bands, widening of the mesenteric base, repositioning the small bowel to the right and the colon to the left, and appendectomy, with the goal of preventing volvulus and future obstructive events. With advances in minimally invasive surgery, laparoscopic Ladd has become an increasingly feasible option, particularly in the absence of midgut volvulus (9). The laparoscopic approach offers the advantages of minimal invasiveness and a shorter recovery period. Studies demonstrate that laparoscopic Ladd in adults and adolescents is safe and effective, relieving symptoms and improving quality of life (10). In addition, it is associated with shorter operative and hospital times and a lower rate of postoperative complications, making it an attractive therapeutic choice (11). Given this child's perforated appendicitis and purulent peritonitis, our index operation prioritized source control (appendectomy, lavage, drainage), with planned interval assessment for the need and timing of Ladd based on recovery and follow-up.

In our patient, the index operation prioritized source control in the context of perforated appendicitis with purulent peritonitis. Extensive adhesions, gross contamination, and the lack of obvious omental necrosis led us to manage the omental torsion conservatively. This approach was justified by the subsequent ultrasound, which demonstrated preserved omental perfusion and resolution of edema. In children with partial omental torsion and severe peritonitis, limiting operative time and avoiding additional resection may be reasonable, provided that close postoperative imaging and clinical follow-up are ensured.

When contextualizing this case, we found only a few pediatric reports describing the combination of congenital malrotation, left-sided appendicitis, and secondary omental torsion. Most published pediatric series of omental torsion involve primary right-sided lesions without malrotation. Our case therefore adds to the limited literature and underscores that left-sided omental abnormalities in children should prompt deliberate consideration of malrotation, especially when imaging suggests an atypical location of the appendix or ileocecal region.

This report has several limitations. No intraoperative photographs were captured because of the emergent and contaminated field; a schematic illustration (Figure 3) is therefore used to depict the malrotated anatomy and left-sided appendix. Although this limits direct visual corroboration, the integrated clinical, imaging, and operative data provide educational value and highlight a practical diagnostic pitfall—namely, that left-sided omental abnormalities with appendiceal signs should trigger careful assessment for malrotation in the preoperative differential diagnosis. Longer-term follow-up is also needed to determine whether and when a Ladd procedure will be required to mitigate future volvulus or obstruction risk.

## 4 Conclusion

In children with left-sided appendicitis and omental abnormalities, congenital midgut malrotation should be considered early as the primary anatomic substrate to optimize diagnosis and operative planning. Ultrasound and CT rapidly characterize inflammation and abnormal localization, whereas a UGI series remains the reference test for confirming malrotation in stable patients. In contaminated emergencies, operative priorities should focus on source control, with interval assessment of the need and timing of a Ladd procedure under close clinical and imaging follow-up.

## Data Availability

The original contributions presented in the study are included in the article/Supplementary Material, further inquiries can be directed to the corresponding authors.
